# Characteristics of Fishing Operations, Environment and Life History Contributing to Small Cetacean Bycatch in the Northeast Atlantic

**DOI:** 10.1371/journal.pone.0104468

**Published:** 2014-08-14

**Authors:** Susie Brown, David Reid, Emer Rogan

**Affiliations:** 1 School of Biological, Earth and Environmental Sciences, Cork Enterprise Centre, University College Cork, Distillery Fields, North Mall, Cork, Ireland; 2 Marine Institute Headquarters, Rinville, Oranmore, Galway, Ireland; Monash University, Australia

## Abstract

Fisheries bycatch is a key threat to cetacean species globally. Managing the impact requires an understanding of the conditions under which animals are caught and the sections of the population affected. We used observer data collected on an albacore tuna gillnet fishery in the northeast Atlantic, to assess operational and environmental factors contributing to bycatch of common and striped dolphins, using generalised linear models and model averaging. Life history demographics of the captured animals were also investigated. In both species, young males dominated the catch. The age ratio of common dolphins was significantly different from that estimated for the population in the region, based on life tables (G = 17.1, d.f. = 2, p = 0.002). Skewed age and sex ratios may reflect varying vulnerability to capture, through differences in behaviour or segregation in populations. Adult females constituted the second largest portion of the bycatch for both species, with potential consequences for population sustainability. Depth was the most important parameter influencing bycatch of both species and reflected what is known about common and striped dolphin habitat use in the region as the probability of catching common dolphins decreased, and striped dolphins increased, with increasing depth. Striped dolphin capture was similarly influenced by the extent to which operations were conducted in daylight, with the probability of capture increasing with increased operations in the pre-sunset and post-sunrise period, potentially driven by increased ability of observers to record animals during daylight operations, or by diurnal movements increasing contact with the fishery. Effort, based on net length and soak time, had little influence on the probability of capturing either species. Our results illustrate the importance of assessing the demographic of the animals captured during observer programmes and, perhaps more importantly, suggest that effort restrictions alone may not be sufficient to eradicate bycatch in areas where driftnets and small cetaceans co-occur.

## Introduction

Anthropogenic pressures on the marine environment are diverse and complex and include pollution, habitat disturbance, invasive species introductions, human induced climate change and fishing [1]. Whilst climate change and fishing pressure are acknowledged as being the main influences on marine ecosystems [2], overfishing has a significant impact on marine exploited communities [1], can act to increase the impact of climate change [3], [4], and continues to be viewed as the key anthropogenic impact affecting marine ecosystems [5]. The pressures placed upon the marine environment continue to grow and as a consequence the need to improve marine management practices has become more urgent [6].

Many large top predators have been subjected to overfishing [7], contributing to the decline and collapse of target species around the world [1], [7]. Alongside target species impacts, the capture of non-target or “bycatch” species [8] ensures commercial fishing is a key driver affecting the biodiversity of marine ecosystems, the loss of which is increasing on a global scale [9]. Bycatch of marine mammals [10], [11], seabirds [12], elasmobranchs [13] and reptiles [14], has been documented in fisheries around the world [15], in extreme cases exceeding target catch [16], [13] and resulting in declines of some species [11]. Management decisions must consider, amongst other factors, the risk posed by fishing to non-target species [2], [12], [17], [18], [19].

Impact on non-target species was a key reason behind restrictions on the use of driftnets by fleets in the European Union. The indiscriminate nature of driftnets and resulting high volume bycatch of non-target species [20] led the Council of the European Commission to restrict the length of driftnets targeting highly migratory species in 1991, culminating in a ban on use of the gear to target such species in 2002 [21]. Driftnets, for large pelagic species, operate legally in regions outside the EU, including the US Pacific, and are subject to strict management regimes which seek to monitor and mitigate marine mammal bycatch, through the use of season and area closures, observer programmes and acoustic devices such as “pingers” [22], [23], [24]. Driftnet operations in the southwest Atlantic are subject to much less regulation [25] and in the EU large scale driftnets reportedly operate illegally in the Mediterranean [26]. High numbers of marine mammals, seabirds, turtles, and non-target fish species such as sharks, occur as bycatch in driftnets [20], [27]. Cetacean species are thought to be particularly vulnerable to the effects of bycatch due to the relatively low abundance of their populations, and because their long life spans, late maturation and low reproductive rates limit the capacity of their populations to recover from such pressures [28]. Successful management of the impacts of driftnets on cetaceans requires an understanding of the conditions under which animals are caught and the species and life history stages impacted.

Observer programmes document the cetacean species taken by fisheries and facilitate assessment of the number of species removed by that fishery. The resulting datasets can provide additional information regarding the operational and environmental characteristics of the capture events and, at times, the characteristics of the animals caught. In 1996, observers accompanied vessels of the Irish fleet deploying surface driftnets targeting albacore tuna (*Thunnus alalunga*) in the Bay of Biscay and Celtic Sea. Focusing on small cetaceans, we examined the resulting dataset with the aim of assessing the relative influence of a number of operational and environmental factors on cetacean bycatch. Additionally, the life history characteristics of the captured animals were assessed, with the objective of determining whether particular species, sexes or age groups were more prone to incidental capture. The objectives of this study were to identify the sections of the population potentially most at risk and the operational and environmental factors associated with small cetacean bycatch.

## Methods

### Observer programme

**Figure 1:**
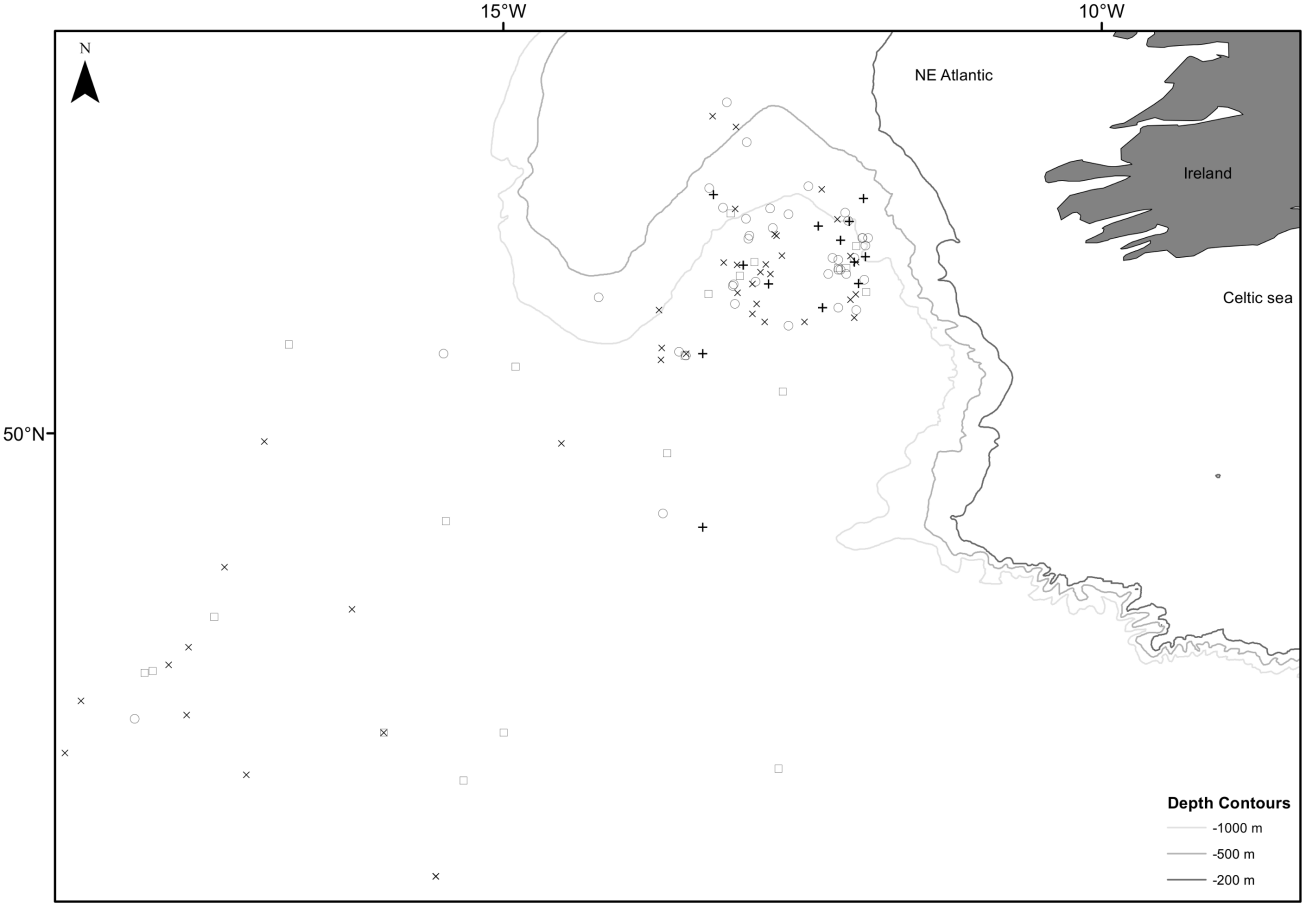
Location of fishing operations and bycatch events. Zero bycatch of common or striped dolphin (x symbol); common dolphin bycatch (empty circle); striped dolphin bycatch (empty square); common and striped dolphin bycatch (+ symbol).

Observer coverage extended to 120 of an estimated 261 hauls (Figure 1). The number and species of cetacean, fish, seabirds and turtles caught, landed and discarded were recorded. This paper examines the small cetacean bycatch only. Full details of the observer programme procedures, methodologies and results are presented in [27].

Observers recorded a number of operational parameters relating to each set. The nets deployed were monofilament nylon twine with stretch mesh size of 17.8 cm. Additional operational parameters recorded included the length of net deployed; the depth of net deployed (based on the number of meshes); the time of setting and hauling; the length of time taken to set and haul the net; and soak time (calculated as the duration between half the time taken to set the net and half the time taken to haul it). Environmental variables recorded included latitude and longitude of net position; depth of water column; sea state (Beaufort scale) during setting and hauling of nets; and the date of the fishing operation.

Where possible, within the constraints of time and space, cetaceans were brought on board the fishing vessel for further examination. Life history parameters were collected including species; sex and total length (tip of beak to notch in tail fluke). Post-mortem examinations were carried out on a number of animals and biological samples, including teeth, were collected.

### Life history traits

Common dolphin (*Delphinus delphis*) and striped dolphin (*Stenella coeruleoalba*) dominated the cetacean bycatch and were subject to life history assessments. Age was estimated for 69 of the 150 common dolphins and 47 of the 66 striped dolphins using teeth (full details of the methodology used to estimate age via teeth are presented in [29]). For the remaining animals, age was estimated using the nose to tail length of the animals aged using teeth, informed by published length at age data [29], [30], [31], [32]. Aged individuals were classified, by sex, into one of three broad age groups: juvenile; sub-adult; and adult (Table 1). Age groups were based broadly on growth phases and the approximate age when sexual maturity is reached [29], [30], [31], [32].

**Table 1:** Age group classification of male and female common and striped dolphins determined through analysis of teeth and age at length data.

Species	Sex	Group	Age	Length	Source of supporting information
**Common dolphin**	Male	Juvenile	≤4	≤185 cm	[30]
		Sub-adult	5–9	186–199 cm	
		Adult	≥10	≥200 cm	
	Female	Juvenile	≤4	≤172 cm	[29], [31]
		Sub-adult	5–7	173–184 cm	
		Adult	≥8	≥180 cm	
**Striped dolphin**	Male	Juvenile	≤4	≤185 cm	[32]
		Sub-adult	5–9	186–210 cm	
		Adult	≥10	≥210 cm	
	Female	Juvenile	≤4	≤170 cm	[32]
		Sub-adult	5–10	171–195 cm	
		Adult	≥11	≥195 cm	

To test the hypothesis that males and females were equally likely to be caught, sex ratios, for each species, were compared to a theoretical 1∶1 ratio using a G test. To test the hypothesis that the age composition of the bycatch animals was similar to that of the population in the region, the proportion of each age group in the northeast Atlantic was estimated using published information on survivorship (l*_x_*) in female common dolphins from the Bay of Biscay [28] under the assumption that survivorship would be similar between the two neighbouring areas. Survivorship was used to estimate the proportion of animals dying at each age (d*_x_*) based on the following equation
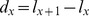
Proportion dying (d*_x_*) was used to estimate the proportion of the population in each of the three age groups which was expected to die. This was then compared to the proportions seen in the bycatch. The analysis was extended to examine the age ratios of the male common dolphins using the survivorship data for females, based on the assumption that there would be no difference in survivorship between the sexes. It was not possible to examine the age ratios of striped dolphins due to a lack of published life tables or similar data. G tests were used to make all comparisons and all analysis was conducted in software package R [33].

### Environmental and operational variables

The influence of operational and environmental variables on the occurrence of cetacean bycatch was assessed. Separate assessments were conducted for the occurrence of common dolphins and striped dolphins. Observers accompanied each vessel on a number of trips and between 3 and 10 sets were observed per trip, therefore the resulting data had a hierarchical structure. Sets took place on consecutive days, potentially introducing temporal correlation. The presence/absence of each species was initially assessed by fitting general estimating equations (GEE) [34], with an autoregressive correlation structure, under the geepack package [35], [36], [37]. A variable “VesselTrip” was created and included in the models to identify observations from the same fishing trip by the same vessel. The correlation of the bycatch between sequential hauls within the same vessel trip was low for both common (0.05) and striped dolphin (0.09); therefore final model fitting was with generalised linear mixed modelling (glmm), with VesselTrip fitted as a random effect, under the glmmML package [38].

Variables included in the models of common and striped dolphin occurrence were selected *a priori* from the parameters collected by the observers. The variables were checked for collinearity using pairwise Spearman’s Rank correlation coefficients, with a correlation coefficient greater than 0.5 indicating variables were proxies for each other [39]. Five variables remained in the analysis when collinear variables were removed; depth, sea state, moon, daylight and effort. Eight collinear variables were removed from the assessment; latitude and longitude (correlated with depth); date of net set and haul (correlated with moon illumination); time of net set and haul (correlated with daylight); and set and haul duration (correlated with soak time which was included in calculating effort).

Depth (environmental variable) was used to describe the depth of the water column where the fishing operation took place. Sea state (environmental variable) described the maximum sea state (Beaufort scale) recorded during net set and haul. Moon (environmental variable) described the percentage illumination of the moon the night of the fishing operation. Daylight (operational variable) described the proportion of the set and haul occurring in daylight i.e. pre sunset and post sunrise. All nets were soaked at night but there was variation in the timing of net setting and hauling and therefore the extent to which these operations were conducted prior to sunset and post sunrise. The time taken to set nets ranged from 30 to 235 minutes and hauling time ranged from 150 to 660 minutes. Typically net setting began several minutes before sunset (mean 32 minutes before sunset, range 1–113 minutes) with only 23 net sets beginning after sunset. Net hauling continued after sunrise in every case (mean 270 minutes after sunrise, range 11–607 minutes). Effort (operational variable) described the length of the net deployed multiplied by soak time, where soak time was calculated as the time between the halfway point in the set and the halfway point in the haul. The explanatory variables were standardised to have a mean of zero and a standard deviation of one prior to analysis.

All possible model permutations were created and model selection was based on Akaike’s Information Criterion AIC [40]. Models were ranked by AIC value and the model with the lowest AIC was considered the most parsimonious within the suite of models. Models within 2 ΔAIC of the most parsimonious model were considered to be similar in their empirical support and were included in the set of candidate models [41]. Akaike weights *w_i_* were calculated for each model and represented the proportional chance of that model being the best model within the set of candidate models [41]. The relative importance (RI) of each variable in determining the occurrence of the species in the bycatch was determined by summing Akaike weights over all candidate models containing the explanatory variable and ranged from 1 (most important) to 0 (least important) [41]. Since model comparisons resulted in a number of models similar in empirical support, we conducted model averaging across all models within 2ΔAIC of the most parsimonious model [41]. Model averaged parameter estimates were produced for each variable, with unconditional standard errors incorporating model uncertainty. Model averaging was conducted using the “MuMIn” package [42]. All analyses were conducted in software package R [33].

### Ethics statement

In Ireland, all cetacean species are protected under the Wildlife (Amendment) Act 1976–2005, therefore sampling was conducted under permit issued by the National Parks and Wildlife Service, Department of Arts, Heritage and the Gaeltacht. The study was entirely based on data collected post-mortem from cetacean carcasses bycaught in an albacore tuna fishery operating in the north east Atlantic. Sampling was conducted on-board fishing vessels and took place over a large area from 46° to 52°N and 11° to 18°W. Sampling did not involve observation or experimentation on live animals or captive animals, therefore ethical approval was not required.

## Results

### Life history characteristics

Cetacean bycatch was recorded in 79 of the 120 observed hauls with 242 individual cetaceans recorded across eight species. Common dolphin and striped dolphin were the most frequently occurring species with 150 common dolphins recorded across 51 sets and 66 striped dolphins recorded across 35 sets (as reported previously [27]). Other species captured were Atlantic white-sided dolphin (*Lagenorhynchus acutus*), Risso’s dolphin (*Grampus griseus*), long-finned pilot whale (*Globicephala melas*), minke whale (*Balaenoptera acutorostrata*), bottlenose dolphin (*Tursiops truncatus*) and sperm whale (*Physeter macrocephalus*). Single individuals were caught in 23 sets with 56 sets having more than one individual. Multiple species were caught together in 18 sets. Common dolphin and striped dolphin were caught together in 13 sets.

**Figure 2:**
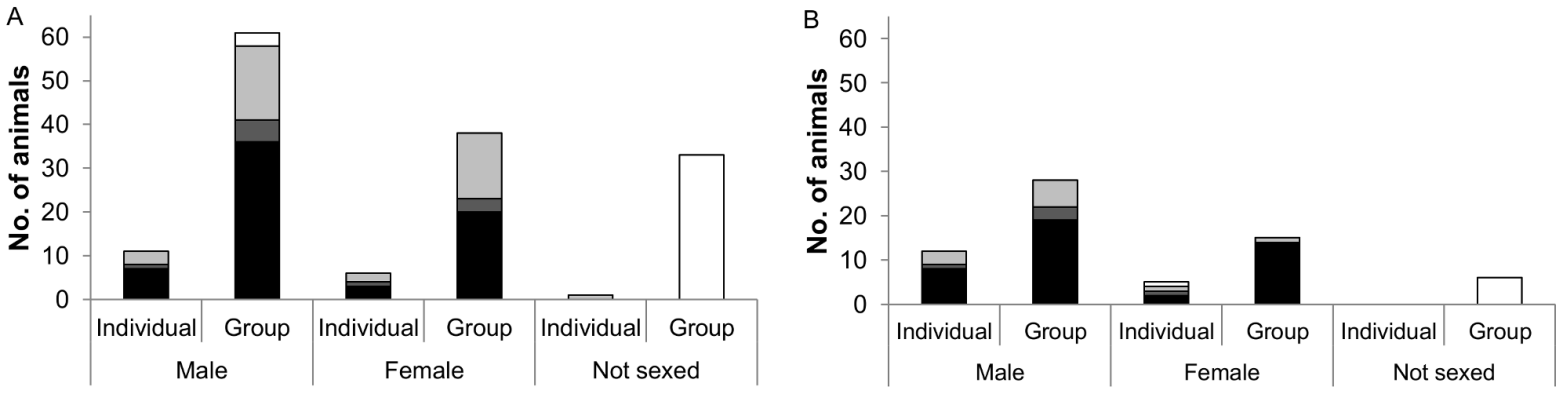
The age group and sex of A) common and B) striped dolphins caught as individuals, or as part of groups. Juvenile (black), sub-adult (dark grey), adult (light grey), not aged (white).

Sex was determined for 116 common dolphins and the majority (n = 72) were male. The sex ratio was 1.64∶1 and significantly different from the 1∶1 ratio which would be expected if males and females were equally likely to be caught (G = 9.04, d.f. = 1, p = 0.002). Age was determined for 113 individuals and 66 were juveniles (43 male and 23 female), 10 were sub-adults (6 male and 4 female) and 37 were adults (20 male and 17 female) (Figure 2A). The age ratio of female common dolphins was significantly different from that estimated for the female population in the north east Atlantic, as determined from life tables (G = 17.1, d.f. = 2, p = 0.002) with more juveniles and fewer sub-adults caught than were estimated to be present in the population. This was also the case for male common dolphins (G = 32.68, d.f. = 2, p<0.0001) although it must be stressed that this analysis was based on a life table for female common dolphins in the absence of the equivalent for males.

Common dolphins were most frequently captured in groups, with 33 sets catching multiple individuals and 18 sets capturing single individuals, including 6 sets which captured a single common dolphin alongside one or more individuals of another species. The average group size was 4 individuals, with the largest group catch being 13 individuals. The term “group” refers to an individual caught alongside at least one individual of the same species. It was not possible to tell whether individuals came from the same social groups. Time and space for processing animals was limited, therefore it was not possible to ascertain the sex of every individual within group catches. Sex was not determined for 33 of the 132 common dolphins which were caught in groups. The majority which were sexed were male (n = 61), with 38 female (Figure 2A). Mixed sex groups made up the bulk of group catches (n = 18) and mixed sex groups were most frequently made up of a mixture of juveniles and adults (n = 8). All male groups were the second most common (n = 8) (Table 2). The age composition of group catches was dominated by groups of adults and juveniles (n = 13) and these groups were predominantly mixed sex (n = 10).

**Table 2:** The age class and sex composition of common dolphin and striped dolphin group catches.

	Common dolphin		Striped dolphin	
	No. of groups	No. individuals within groups	No. of groups	No. individuals within groups
**All juveniles**	4	12	7	19
**All sub-adult**	0	0	0	0
**All adult**	3	6	0	0
**Mix of juvenile & sub-adult**	2	7	1	5
**Mix of juvenile & adult**	13	56	6	16
**Mix of sub-adult & adult**	1	3	0	0
**Mix juvenile, sub-adult & adult**	2	9	1	3
**Not aged**	8	39	3	6
**All male**	8	19	5	8
**All female**	1	2	0	0
**Male & female**	18	85	11	35
**Not sexed**	6	26	3	6

Sex was determined for 60 of the 66 striped dolphins caught. Twice as many of the sexed individuals were male (n = 40) (Figure 2B) and the sex ratio was significantly different from a 1∶1 ratio (2∶1) (G = 6.79, d.f. = 1, p = 0.009) again suggesting that males were more likely to be caught than females. Age was determined for 59 individuals and 43 were juveniles (27 male and 16 female), 5 were sub-adult (4 male and 1 female) and 11 were adult (9 male and 2 female) (Figure 2B). It was not possible to test if these ratios were similar to that of the wider population of striped dolphins owing to the lack information on survivorship for the species.

Striped dolphins were caught as single individuals in 17 sets and as part of a group in 18 sets. The single individual catches included seven cases where a single striped dolphin was captured alongside at least one individual of another species. Groups were smaller than for common dolphins with an average group size of 2.7 and the largest group comprised five individuals. As with common dolphins the majority of animals occurring in group catches were male (n = 28) with close to half as many females caught (n = 15). Again, similar to common dolphins, mixed sex groups dominated the group catches (n = 11), with 6 of the mixed species groups composed entirely of juveniles. All male groups were the second most frequently captured group (Table 2). The age composition of group catches differed from that of common dolphins with catches of adults and juveniles (n = 6) occurring almost as frequently as all juvenile groups (n = 7) (Table 2).

### Operational and environmental parameters

The most parsimonious model for common dolphin occurrence contained the variable depth. The variables sea state, light and moon also featured in the set of candidate models (models with ΔAIC ≤2 of the most parsimonious model) (Table 3). On the basis of model averaging, the relative importance of the variables in determining the occurrence of common dolphin bycatch was, in order of importance; depth; sea state; daylight; and moon (Table 4). The relative importance of depth and its occurrence in the most parsimonious model indicates that, of the variables examined, the depth of the water column where the fishing operation took place was the most important predictor of common dolphin bycatch. The probability of catching common dolphins decreased with increasing depth. Sea state and daylight had a similar level of importance. The probability of common dolphin bycatch increased with increasing sea state during setting and hauling of nets, and with increased time spent setting and hauling pre-sunset and post-sunrise. Moon illumination had very little importance in common dolphin bycatch with the probability of common bycatch occurring decreasing with increasing moon illumination. Effort did not feature in the set of candidate models for common dolphin bycatch.

**Table 3:** Candidate models (ΔAIC≤2) of common dolphin occurrence in bycatch in the albacore tuna fishery.

Rank	Model structure	AIC	ΔAIC	*w_i_*
**1**	Depth	141.22	0.00	0.29
**2**	Depth+Sea state	141.65	0.43	0.24
**3**	Depth+Light	141.95	0.72	0.21
**4**	Depth+Light+Sea state	142.69	1.46	0.14
**5**	Depth+Moon	142.99	1.76	0.12

**Table 4:** Model averaging results of common dolphin occurrence in bycatch in the albacore tuna fishery.

Variable	β	SE	RI
**Depth**	−1.08	0.27	1.00
**Sea State**	0.26	0.21	0.38
**Daylight**	0.24	0.21	0.35
**Moon**	−0.14	0.22	0.12

β model averaged coefficients; SE standard error; RI relative importance.

The most parsimonious model for the occurrence of striped dolphins contained the variables depth and daylight (Table 5). The variables effort and moon also featured within the set of candidate models. The relative importance of the variables in determining the occurrence of striped dolphins was daylight; depth; moon; and effort (Table 6). The relative importance of depth and daylight and their occurrence in the most parsimonious model indicate that the depth of the water column where the fishing operation took place, and the extent to which fishing operations were conducted during daylight hours, were the most important predictors of striped dolphin bycatch. Daylight and depth had similar levels of importance. As with common dolphin bycatch, the probability of catching striped dolphins increased with increased time spent setting and hauling nets pre sunset and post sunrise. However, contrary to the pattern seen in common dolphin bycatch the probability of catching striped dolphins increased with increasing depth. Effort and moon had relatively low importance. Striped dolphin bycatch increased with effort but decreased with increasing moon illumination. The variable sea state did not feature in the candidate models for striped dolphin bycatch.

**Table 5:** Candidate models (ΔAIC≤2) of striped dolphin occurrence in bycatch in the albacore tuna fishery.

Rank	Model structure	AIC	ΔAIC	*w_i_*
**1**	Depth+Daylight	141.53	0.00	0.36
**2**	Depth+Effort+Daylight	142.81	1.28	0.19
**3**	Depth+Moon+Daylight	143.12	1.59	0.16
**4**	Daylight	143.25	1.72	0.15
**5**	Depth	143.52	1.99	0.13

**Table 6:** Model averaging results of striped dolphin occurrence in bycatch in the albacore tuna fishery.

Variable	β	SE	RI
**Daylight**	0.47	0.27	0.87
**Depth**	0.42	0.23	0.85
**Effort**	0.23	0.24	0.19
**Moon**	−0.17	0.22	0.16

β model averaged coefficients; SE standard error; RI relative importance.

## Discussion

Common and striped dolphins dominated cetacean bycatch in the albacore tuna fishery. With the exception of harbour porpoise (*Phocoena phocoena*), which typically occur in shallower waters beyond the range of the tuna fishery, common dolphins are the most abundant small cetacean in the north east Atlantic in summer, and striped dolphins the second most abundant [43], [44], [45]. Common dolphins are present in both shelf and deep waters in the northeast Atlantic, whilst striped dolphins are restricted to deeper waters [44], [46]. Of the variables examined, the depth of the water column where the fishing operation took place was the most important factor driving the occurrence of each species in bycatch in the albacore tuna fishery. The direction of influence reflects what is known about the distribution of the species across the depth gradient with striped dolphins more likely to be caught in deeper waters and common dolphins in shallower waters. Common dolphins have been recorded as bycatch in mobile [47], [48], [49] and static gears [45], [50] in the northeast Atlantic. Although their abundance in the region and their distribution across the continental slope and deeper offshore waters is likely the key factor accounting for their prevalence in bycatch, as indicated by the importance of fishing depth, behavioural factors and fine scale movements within the region, may underpin the observed patterns.

The bycatch demographics were skewed towards males and young animals for both species, although a number of mature females were also caught. The prevalence of males and young animals in the bycatch suggests that some sections of the common and striped dolphin populations may be more vulnerable to capture than others. Several factors may drive the patterns observed in the bycatch. Firstly, the age and sex structure in the bycatch represents that of the wider population. Secondly, there is age and sex segregation in the wider population leading to different ratios in the area where the fishery operated. Thirdly, behavioural differences between age groups and sexes contributed to sections of the population being more vulnerable [51]. Finally, the size selective nature of gillnets contributed to the frequency of smaller animals in the bycatch. A single driver may be responsible for the observed pattern, but given the resultant complexities of multiple drivers, potentially acting in parallel, it is difficult to determine which scenario has greatest influence.

There is a lack of unbiased sources of demographic data for cetaceans [28] and little is known of the age and sex structure of the wider striped and common dolphin populations in the northeast Atlantic, as a whole. Delphinidae populations are, in general, segregated by age and sex [52] and skewed sex and age ratios in stranded and bycatch animals supports the hypothesis of age and sex segregation in the wider population of the northeast Atlantic, leading to regional variation in age and sex structure. The age distribution of common dolphins in this study is similar to the pattern in strandings along the French coast [28] but differs from that reported in pair trawls in northwest Spain, where animals in the sub-adult range were caught in greater numbers than juveniles and adults [47]. The prevalence of common dolphin males has been recorded in other gillnet fisheries [53] and in trawls [47]. Striped dolphin males dominated in Spanish driftnets in the western Mediterranean, with common dolphins exhibiting a more even sex ratio [54]. Whilst, the male bias in common dolphins recorded in trawlers operating off northwest Spain resulted from several large all male capture events and was thought to provide evidence of groups of bachelor males in the area [47]. When groups of mixed age where captured in the albacore tuna fishery they were most frequently composed of adult females with juveniles, indicating that the fishery may have overlapped with calving grounds or maternal feeding grounds [31]. The overlap between the albacore fishing season and the common dolphin calving season [29], [30] provides further support to the theory that the fishery encountered these age groups more frequently than any other contributing to their abundance in bycatch.

Whilst the skewed age and sex distributions recorded in the bycatch are interesting, it is important to consider that not all individuals were aged and sexed. This was particularly true in the case of group catches where post mortem examination was constrained by the time and space available to process large numbers of animals on board. Furthermore, we based our assessment of common dolphin population age structure on survivorship of females in the Bay of Biscay as determined through strandings [28]. Aside from the biases inherent in demographic data from stranded animals [28], the albacore fishery operated in waters north of the Bay of Biscay, off the western coast of Ireland. We assumed, for the purposes of the assessment, that survivorship was similar between these regions. Given the proximity of the regions and the apparent lack of genetic structure within the common dolphin population in the northeast Atlantic [55] this assumption seems reasonable. However, analysis of heavy metals [56], [57] and stable isotopes [57] suggest the existence of a neritic stock on the continental shelf and an oceanic stock offshore. The albacore fishery operated in offshore waters and it is likely that bycaught common dolphins predominantly came from the oceanic stock. Conversely, strandings are potentially dominated by animals from the neritic stock [28]. Furthermore we assumed, for the purposes of the assessment, that survivorship was similar for males and females as data on the survivorship of males was not available. Given the prevalence of young male bycatch in the albacore tuna fishery and other fisheries operating in the northeast Atlantic [47], [53] it is possible that survivorship in male common dolphins is lower than that of their female counterparts in the region.

Common and striped dolphins form social groups of different age classes so it is perhaps not surprising that group catches outnumbered incidental takes of single individuals throughout the range of the fishery. It is not possible to determine whether group catches were truly social groups, or whether other factors resulted in multiple individuals being caught in the same set. Nevertheless, the occurrence of both individual and group catches could suggest that some individuals within a social group, such as young animals, may be more vulnerable to capture than others, or may behave differently from other individuals within the group. These potential differences in behaviour between age groups and sexes may compound spatial segregation within populations. Behavioural responses to boats may contribute to bycatch as both species have been documented to respond to survey boats by approaching them [43], [44], [46]. A similar response elicited by fishing boats setting and hauling nets may increase the risk of being caught. Across all mammal populations, males and adolescents are less risk averse than females and other age groups [58], [59] and may be more inclined to approach a boat during the setting or retrieval of gear, increasing the risk of capture. Sub-adults, or adolescents, were under represented in the albacore tuna bycatch, which in addition to the hypothesis of age and sex segregation amongst the population could indicate an influence of learned behaviour or experience. The younger animals, which dominated the bycatch, may have less precise echolocation abilities [31], may vary in their behavioural response to boats or may lack the physical skills or experience necessary to feed within the vicinity of the net without becoming entangled.

Gillnets are size selective and catch typically reflects stock structure and the mesh size deployed [60]. To our knowledge the influence of size selectivity on cetacean bycatch in gillnets has not been tested with regard to mesh size. Measures to reduce selectivity towards cetaceans typically focus on modifications to gear deployment and retrieval, or changes to fishing practices such as the height of static gear deployment in the water column, or the speed, depth and duration of trawls [17]. It is possible that mesh size also contributes to selectivity for cetaceans and may act to compound the influence of sex and age segregation and behaviour. The stretch mesh size of deployed gillnets was 17.8 cm and the majority of the common and striped dolphin bycatch were juvenile animals less than 185 cm in length. Mean length of juvenile bycatch was 136 cm for common dolphins and 146 cm for striped dolphins. In a size selectivity experiment for sandbar shark (*Carcharhinus plumbeus*), all mesh sizes captured all size classes but maximum selectivity of 17.8 cm mesh was between 95 and 124 cm size classes [60]. Sharks and dolphins have the same fusiform shape, and size selectivity may be similar. However, features of dolphin morphology, including long, narrow rostrums may increase the vulnerability of larger individuals to capture, and this may explain why adult dolphins were also captured in large numbers. Cetacean bycatch in the albacore fishery also included larger species which lack long, narrow rostrums, including minke whales, long-finned pilot whales and sperm whales and it is possible that the mechanism by which larger individuals, and larger species, are captured differs from that of smaller animals. It was hypothesised that in the case of sandbar sharks, larger animals got caught by wrapping themselves in the net, or breaking it and becoming caught in larger openings. A similar mechanism of entanglement may capture larger cetacean species and larger, older, common and striped dolphins.

The behaviour of dolphins around nets, and the mechanism by which they become entangled, is not fully understood but assessment of echolocation abilities and observations at sea suggest some species may be capable of detecting and avoiding nets [61], [62]. A number of scenarios could explain how cetaceans become entangled in gear they are capable of detecting. It has been suggested that the animals may not echolocate while travelling, failing to detect the net while in transit; they may detect the presence of the net but not identify it as a barrier; they may become distracted while feeding in the vicinity of the net; or fish entangled in the net, and free swimming in its vicinity, may block detection of the net [61], [62], [63]. Common dolphin bycatch increased with increasing sea state during setting and hauling suggesting that increasing turbidity, resulting from increased sea state, may hamper net detection.

The timing of cetacean bycatch within a fishing operation is also poorly understood. Video technology may be useful for monitoring stages which cannot be observed from deck for mobile gears [64] but may not be appropriate for static nets. The operational variable effort, which described the length of net deployed and its soak time in the water, had little influence on the occurrence of striped dolphin and no influence on the occurrence of common dolphin bycatch. The variable moon illumination, which captured the brightness of the moon during the soak, also had very little influence. The importance of operational variables relating to the setting and hauling of the net, including the influence of sea state during setting and hauling on common dolphin bycatch and the importance of daylight during setting and hauling for both species, suggests that this may be the stage of the fishing operation when the species are most vulnerable.

A greater vulnerability towards fishing operations conducted at night has been documented in a number of fisheries operating in the northeast Atlantic [45], [47], [48], [65] and our findings suggest that timing of operations could be important on a much finer scale. The increase in bycatch with increased daylight during setting and hauling may seem counter intuitive as visual acuity and therefore visual detection of the net should be greater in daylight than in darkness. Indeed, although illumination of the moon had comparatively little influence on the occurrence of either species, the probability of bycatch decreased with increasing lunar illumination. Two mechanisms may contribute to increased likelihood of cetacean bycatch during setting and hauling in daylight. Firstly, observers monitoring nets during daylight hours may be more likely to document animals which fall out of the net as it is being hauled on board. These “drop outs” could be missed during operations occurring in darkness if the animal fell out some distance from the boat. Likewise, carcases floating in the water are more likely to be spotted and documented during daylight than at night. Secondly, the species may be more likely to come into contact with the net during certain periods of the night as a result of diurnal behaviour or movement.

Little is known about the behaviour or movements of common and striped dolphins, worldwide. Observations from the Mediterranean [66], [67], [68] and Australia [69] may shed light on how diurnal movements of these species may contribute to their capture. Feeding in striped dolphins, in the French Riviera, was found to peak in the three hours before sunset and after sun rise [66]. The same study revealed that although the majority of animals remained in offshore areas a significant number of animals exhibited diurnal movements from inshore to offshore areas coinciding with these peaks in feeding activity. If similar patterns of feeding and movement are present in striped dolphins in the northeast Atlantic, operations conducted during the pre-sunset and post sunrise period could coincide with these activity peaks, thus encountering more animals. Although diurnal patterns in feeding behaviour have been detected in common dolphins [68], [69], similar diurnal horizontal movements have not. Feeding in oceanic common dolphin peaks at dusk, as the animals feed on mesopelagic fish which migrate to the surface at night [70]. Common dolphins have been recorded appearing in the vicinity of prawn trawlers, in Australia, during net hauling, seemingly attracted by the sound of the engines [71]. The extent of setting and hauling occurring in daylight may be less important for common dolphins if the overall driver for their presence at the boat is their attraction to it, rather than any increase in horizontal movements during this period.

The complex nature of species distribution and the dynamic nature of the fishery in space and time mean it is an oversimplification to suggest that our model captures all variables driving bycatch of these species. Observer data have been used to examine the influence of operational and environmental variables in cetacean bycatch for a number of species and fisheries e.g. [64], [72], [73], [74], [75] and although observer data are important for examining the patterns in bycatch, several issues should be considered. There is the potential for boats carrying observers to vary their behaviour to reduce the likelihood of bycatch occurring, perhaps by avoiding areas where it has occurred in the past [28], however in the northeast Atlantic fishing for albacore occurs between June and October and the short duration of the albacore season, and of the fishing trips, makes avoidance less likely in this case. Secondly, whilst bycatch “hotspots” occur in many regions it is worth noting that lack of documented bycatch incidents in an area, does not mean that bycatch would not happen there under different circumstances [76] and this is particularly relevant when assessing the role of operational and environmental factors.

The lack of importance of effort in bycatch occurrence, considered alongside the importance of depth, indicates that the location of the net, rather than the length of the net or time in the water, influenced the probability of cetacean bycatch occurring in this fishery. If animals are more vulnerable during net setting or hauling, either as a result of diurnal movement, timing of feeding, or attraction to the boat, the length of net deployed would have little influence on numbers captured and restricting net length would have little impact as a mitigation measure. Prior to banning the use of pelagic driftnets to target highly migratory species, the European Commission prohibited the deployment of driftnets over 2.5 km in length. Our results support the decision that length restrictions alone are not sufficient to limit cetacean bycatch. It is also important, in the context of Ecosystem Based Fishery Management (EBFM), to emphasize that the EU restrictions on driftnets were introduced not only to reduce levels of cetacean bycatch but to address the unsustainable level of seabird, turtle and non-target fish species in this gear.

## Conclusion

This study illustrates that data collected during observer programmes can be utilised beyond estimating the number of animals taken by a fishery and that key patterns, in the factors influencing bycatch occurrence, can be elucidated. Analysis of bycatch should not only consider the operational and environmental factors which may drive it but, where possible, should consider the age and sex of the animals taken. Differing susceptibility across age classes and sexes could have important consequences for population recovery, as it may take longer to recover from depletion if animals of reproductive age [77], and particularly females [78], [79] were impacted. Whilst driftnets are no longer deployed to target albacore tuna in the northeast Atlantic, striped and common dolphin bycatch persists in fisheries deploying other gears including pair trawls, otter trawls and set gillnets. Under EBFM, Ecological Risk Assessments for the Effects of Fishing (ERAEF) are increasingly used to examine the impact of commercial fisheries on non-target and bycatch species including cetaceans [19]. ERAEF includes assessment of the ability of a species to withstand fishing pressure and the likelihood that it will encounter fishing pressure based on spatial and temporal overlap with the fishery and susceptibility to the gear deployed. It is our view that observer programmes for all fisheries should, where possible, include detailed assessment of the sections of the populations affected as this, considered alongside the operational and environmental conditions under which bycatch occurs, could contribute to the ongoing refinement of ERAEF in the context of cetaceans.

## Supporting Information

Table S1 **Operational, environmental and bycatch data relating to each net set.**
(XLSX)

Table S2 **Life history characteristics of common and striped dolphin bycatch.**
(XLSX)
